# Supplementary figures and images for: Open-source milligram-scale, four channel, automated protein purification system
Source: PLoS One. 2024 Feb 23;19(2):e0297879. doi: 10.1371/journal.pone.0297879 (PMC10889886; doi:10.1371/journal.pone.0297879)

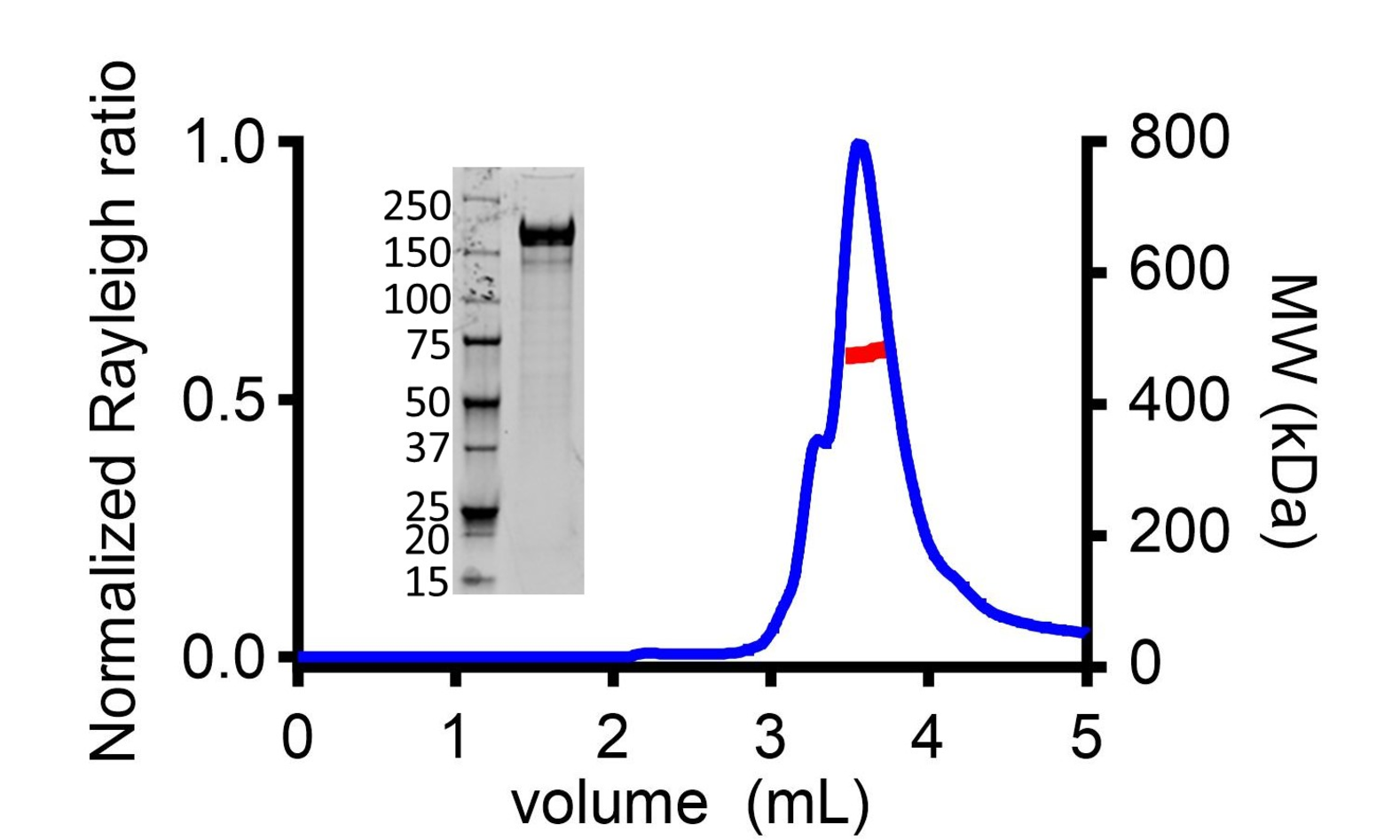

Supplement: S1 Fig — SARS-CoV-2 Spike was purified using a 5 mL HisTrap Excel column on a commercial chromatography instrument (ӒKTA Pure), desalted and concentrated offline, and analyzed by reducing SDS-PAGE (2.5 μg protein loaded) and analytical SEC-MALS. The calculated molecular weight of the major peak is shown in red. (TIF) [file pone.0297879.s003.tif]
